# Child Abdominal Distension Due to a Massive Retroperitoneal Lymphangioma: A Cyto-Histopathological and Clinical Correlation

**DOI:** 10.7759/cureus.72874

**Published:** 2024-11-02

**Authors:** Fabíola P Morais, Iago R Carvalho, Iasmim R Carvalho, Andrea M Luppi, Bruno C Dornelas

**Affiliations:** 1 School of Medicine, Federal University of Uberlandia, Uberlândia, BRA; 2 School of Medicine, UniCerrado, Goiatuba, BRA; 3 Department of Radiology, University of Uberlândia Clinical Hospital, Empresa Brasileira de Serviços Hospitalares (EBSERH), Uberlândia, BRA; 4 Department of Pathology, University of Uberlândia Clinical Hospital, Empresa Brasileira de Serviços Hospitalares (EBSERH), Uberlândia, BRA

**Keywords:** cystic retroperitoneal mass, lymphatic malformation, lymphatic vessels, retroperitoneal lymphangioma, retroperitoneal neoplasms

## Abstract

Lymphangiomas are rare benign tumors that result from lymphatic vessel malformations and/or obstructions commonly on the neck and armpits, being rare in the retroperitoneal space. We report a case of a healthy 25-month-old male with a six-month history of abdominal distension and recurrent episodes of diarrhea who was clinically diagnosed with giardiasis. The complementary evaluation showed a cystic formation occupying the whole abdominal cavity and implanted at the abdominal retroactivity. The patient underwent videolaparoscopic surgery. At histopathological and histochemical examinations, the diagnosis of cystic lymphangioma was stated. The report presents a rare case of retroperitoneal lymphangioma, a condition that is frequently misdiagnosed due to its rarity.

## Introduction

Lymphangiomas are rare benign tumors that result from malformations or the obstruction of lymphatic vessels [1,2] leading to lymphangiectasia. Lymphangiomas account for 5% of all benign tumors in children and often affect the neck (75%) and armpits (20%) [3,4]. Less than 5% of lymphangiomas are intraabdominal sited, where they have been described in the mesentery, gastrointestinal tract, spleen, liver, and pancreas [1,5,6]. Retroperitoneal lymphangiomas make up approximately 1% of all lymphangiomas [4]. They are usually asymptomatic. However, lymphangiomas may present with an abdominal mass, abdominal pain, fever, fatigue, weight loss, nausea, and vomiting, reaching large sizes before becoming symptomatic in most cases [3]. Other clinical presentations include bowel obstruction, sepsis, and disseminated intravascular coagulopathy. Lymphangiomas may rarely present with complications such as infection, rupture, torsion, or bleeding [6].

Approximately 90% of retroperitoneal lymphangiomas are diagnosed prior to two years of age, although they can occur at any age [7]. Spontaneous resolution of lymphangiomas is very rare, so surgical treatment is recommended [8]. Imaging exams are important to detect and characterize the lesion and, therefore, to manage the case [9]. On the other hand, differentiating lymphangiomas from other retroperitoneal cystic tumors only with imaging studies is generally inconclusive, and surgical sampling is required for diagnostic confirmation [10]. In this article, we report the case of a rare and massive retroperitoneal lymphangioma in a child presenting with abdominal distension.

## Case presentation

**Figure 1:**
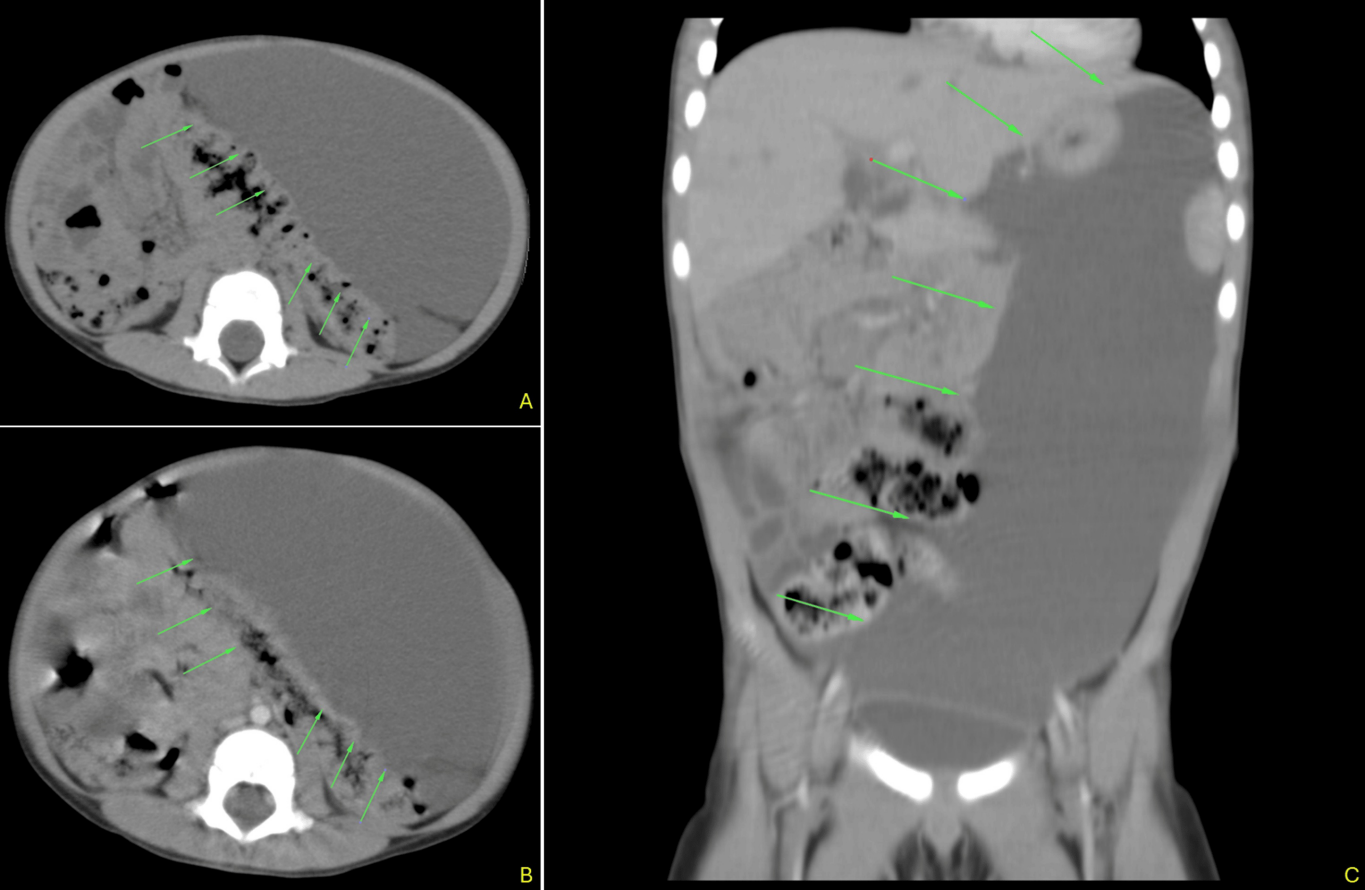
Computed tomography scan images (axial in A and B, coronal in C) demonstrate a massive, homogeneous cystic mass with thin walls in the left hemiabdomen (arrows). The lesion compresses the bowels posteriorly and to the right

A 25-month-old male child with no relevant medical or surgical history was admitted with a six-month history of abdominal distension and intermittent episodes of diarrhea. Clinically, he had been diagnosed with giardiasis and received metronidazole and albendazole. Physical examination revealed a painless, distended abdomen. An abdominal ultrasound was performed and showed a cystic mass occupying the entire abdominal cavity. A CT scan (Figure 1A-C) confirmed the presence of a large and homogeneous cystic tumor in the abdomen with thin walls and dislocating the intestinal loops. No septa nor vegetations were seen.

**Figure 2:**
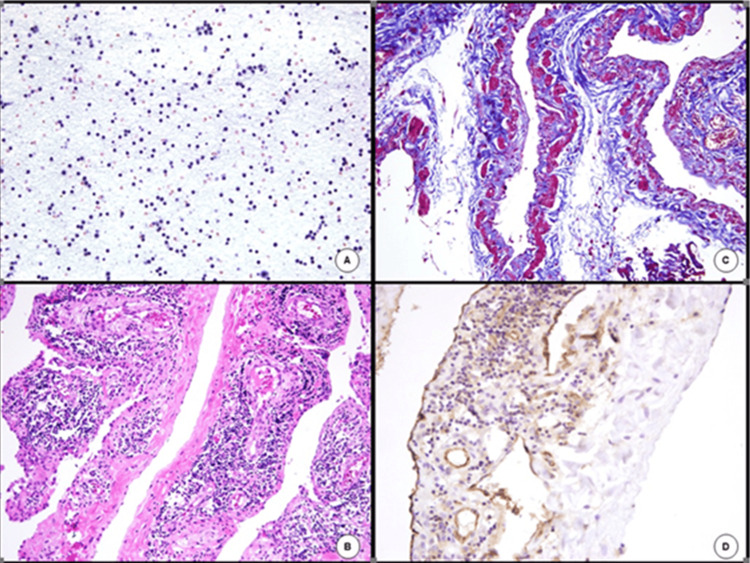
Cytological analysis shows a polymorphous lymphocyte population (A). On histopathological examination, cyst walls are coated by endothelial cells, and the stroma contains a marked lymphocytic infiltration and some smooth muscle bundles (B). Smooth muscle cells stain red by Masson's trichrome (C). The inner lining cells react to CD31 immunohistochemistry staining (D)

A videolaparoscopic surgery was performed, and a massive cyst was seen with its implantation in the abdominal retrocavity and extending to the pelvis. Over 700 mL of clear, straw-colored fluid were aspirated and sent for cytological analysis. Once empty, the cyst was completely removed as it adhered to the small epiploon, pancreas, and transverse colon. Cytological smears were hypercellular and contained a polymorphous lymphocyte population with a predominance of mature lymphocytes consistent with lymphangioma (Figure 2A). Histopathological examination showed large lymphatic channels internally lined up by endothelial cells. The connective stroma contained peripheral lymphoid aggregates and disorganized smooth muscle highlighted by Masson's trichrome (Figures 2B-2C). The coating inner cells showed immunopositivity to CD31. The diagnosis of lymphangioma was stated. The child was discharged two days after surgery, and no recurrences have been recorded.

## Discussion

Lymphangiomas are rare benign tumors of the lymphatic vessels. The majority (>95%) appear in the neck and armpits. Intraabdominal lymphangiomas are uncommon (<1%) and occur more frequently in the mesentery, followed by the omentum, mesocolon, and retroperitoneum [11]. The etiology of lymphangiomas is still unclear but seems to be related to congenital malformation of lymphatic vessels that lead to the sequestration of lymphatic tissue [12] so that abnormal lymphatic channels are dilated, resulting in the formation of uni or multicystic tumors, whose cavity is covered by a layer of endothelium and filled by serous material or kilos [4].

Lymphangiomas are classified as simple (capillary), cavernous, or cystic. Simple lymphangiomas consist of considerable stroline tissue with small lymphatic vases of a thin wall; cavernous are composed of dilated lymphatic vessels, and cystic vessels present as unique or multiple cystic lesions [3,6]. The cystic type is the most commonly found in the retroperitoneum, being asymptomatic in most cases. The most common clinical manifestation is the presence of abdominal mass, which may be associated with pain, weight loss, nausea, and vomiting [13]. The effect of this mass can lead to obstruction and displacement of adjacent organs. However, retroperitoneal lymphangiomas are rarely complicated with ascites, hemorrhage, rupture, torsion, ureteral obstruction, and hematuria [3]. In the case in question, the child presented abdominal distension associated with intermittent episodes of diarrhea.

Differential diagnosis of retroperitoneum cystic lesions includes malignant and benign tumors. Malignant causes stand out: cystic mesothelioma, teratoma, undifferentiated sarcoma, cystic metastases (especially the primary of the ovary and stomach), and malignant mesenchymoma. Benign tumors include lymphangioma, cysts of urothelial and intestinal origin, and microcystic pancreatic adenoma, in addition to other non-neoplastic lesions such as retroperitoneal hematoma, abscess, duplication cyst, ovarian cysts, and pancreatic pseudocyst [14,15]. Based on imaging tests and in the face of numerous hypotheses for retroperitoneal cystic masses, the pre-surgical diagnosis of lymphangiomas is rare [3]. Ultrasound has been the initial technique of choice to identify the lesion and define its cystic structure and tumor size [9]. Sequentially, CT and MRI provide more accurate images regarding tumor extension, location, the involvement of other organs, and the type of fluid within the cystic lesion [6]. Imaging examinations are important to exclude malignancy and establish the anatomical location of the tumor before surgery [1,9].

The treatment of choice for lymphangiomas is surgical excision (laparotomy or laparoscopy) due to the potential for growth and development of complications of these lesions [1,7]. The prognosis after complete resection is excellent. A recurrence rate of 10% has already been described in incomplete resections, as well as malignant transformation, spontaneous regression, and hemoperitoneum stemming from spontaneous hemorrhage [16]. The first description of laparoscopic resection of retroperitoneal lymphangioma was published by Targarona et al. in 1994 [17]. This technique has currently shown several advantages concerning laparotomy, highlighting minimal trauma, pain reduction, and faster postoperative recovery [18]. The final diagnosis of lymphangioma is given by histopathological examination after surgical resection so that the specimen is characterized by the presence of a thin, irregular wall coated with endothelium, containing smooth muscle beams and lymphocyte infiltrate [19]. In the present case, the initial investigation, as recommended, was an ultrasound of the abdomen and, later, CT, which detected a cystic formation in the abdominal cavity. Thus, for further investigation and diagnostic confirmation, the child underwent laparoscopy with complete exéresis of the cystic lesion, and diagnostic confirmation of lymphangioma was given by cytological/histopathological analysis.

## Conclusions

Lymphangiomas are benign tumors believed to result from congenital malformations of the lymphatic vessels, with most cases occurring in the neck and armpits. Retroperitoneal lymphangiomas are rare and typically cystic. They are usually asymptomatic until they reach a significant size, at which point symptoms such as abdominal distension, a palpable mass, abdominal pain, weight loss, nausea, and vomiting may occur.

Diagnosing retroperitoneal lymphangiomas preoperatively through imaging can be challenging due to the variety of differential diagnoses for abdominal cystic lesions, which include both benign and malignant conditions. The use of ultrasonography allows for the identification of the cystic structure and assessment of tumor size, while CT or MRI provides a better understanding of the extent of the condition.

Cytology is a valuable tool, as aspiration of clear, watery fluid further supports the diagnosis and helps distinguish it from other differential diagnoses. Microscopic examination of the smears reveals mature lymphocytes against a proteinaceous background. Based on these findings, a confident diagnosis of cystic lymphangioma can be made through cytology.

## References

[1] Sato T, Matsuo Y, Shiga K, Saito K, Morimoto M, Miyai H, Takeyama H (2015). Laparoscopic resection of retroperitoneal lymphangioma around the pancreas: a case report and review of the literature. J Med Case Rep.

[2] Poroes F, Petermann D, Andrejevic-Blant S, Labgaa I, Di Mare L (2020). Pediatric cystic lymphangioma of the retroperitoneum: a case report and review of the literature. Medicine (Baltimore).

[3] Bhavsar T, Saeed-Vafa D, Harbison S, Inniss S (2010). Retroperitoneal cystic lymphangioma in an adult: a case report and review of the literature. World J Gastrointest Pathophysiol.

[4] Mabrouk A, Ennaceur F, Karoui Y, Nejma EB, Jedidi L, Moussa MB (2022). Giant retroperitoneal lymphangioma in a 70-year-old male: a case report. Pan Afr Med J.

[5] Koenig TR, Loyer EM, Whitman GJ, Raymond AK, Charnsangavej C (2001). Cystic lymphangioma of the pancreas. AJR Am J Roentgenol.

[6] Su T, Li C, Song B, Song D, Feng Y (2023). Case report and literature review: giant retroperitoneal cystic lymphangioma. Front Surg.

[7] Black T, Guy CD, Burbridge RA (2013). Retroperitoneal cystic lymphangioma diagnosed by endoscopic ultrasound-guided fine needle aspiration. Clin Endosc.

[8] Olímpio Hde O, Bustorff-Silva J, Oliveira Filho AG, Araujo KC (2014). Cross-sectional study comparing different therapeutic modalities for cystic lymphangiomas in children. Clinics (Sao Paulo).

[9] Romeo V, Maurea S, Mainenti PP, Camera L, Aprea G, Cozzolino I, Salvatore M (2015). Correlative imaging of cystic lymphangiomas: ultrasound, CT and MRI comparison. Acta Radiol Open.

[10] Gümüştaş OG, Sanal M, Güner O, Tümay V (2013). Retroperitoneal cystic lymphangioma: a diagnostic and surgical challenge. Case Rep Pediatr.

[11] Mohammadi A, Ghasemi-rad M, Abassi F (2013). Asymptomatic lymphangioma involving the spleen and mediastinum in adults. Med Ultrason.

[12] Maghrebi H, Yakoubi C, Beji H (2022). Intra-abdominal cystic lymphangioma in adults: a case series of 32 patients and literature review. Ann Med Surg (Lond).

[13] Mansour S, Kluger Y, Khuri S (2023). Adult primary retroperitoneal lymphangioma: updated facts. World J Oncol.

[14] Yagihashi Y, Kato K, Nagahama K, Yamamoto M, Kanamaru H (2011). A case of laparoscopic excision of a huge retroperitoneal cystic lymphangioma. Case Rep Urol.

[15] Trinh CT, Tran NT, Vo BTT, Van HAT, Hoang VT, Nguyen MD (2021). A case of retroperitoneal lymphangioma in an adult. Hum Pathol Case Rep.

[16] Talukdar S, Alagaratnam S, Sinha A, Thorn CC, Elton C (2011). Giant cystic lymphangioma in childhood: a rare differential for the acute abdomen. BMJ Case Rep.

[17] Targarona EM, Moral A, Sabater L, Martínez J, Luque P, Trías M (1994). Laparoscopic resection of a retroperitoneal cystic lymphangioma. Surg Endosc.

[18] Kang BH, Hur H, Joung YS (2011). Giant mesenteric cystic lymphangioma originating from the lesser omentum in the abdominal cavity. J Gastric Cancer.

[19] Méndez-Gallart R, Solar-Boga A, Gómez-Tellado M, Somoza-Argibay I (2009). Giant mesenteric cystic lymphangioma in an infant presenting with acute bowel obstruction. Can J Surg.

